# Cross-cultural adaptation of the Eating Beliefs Questionnaire into Brazilian Portuguese

**DOI:** 10.1590/1980-549720230011

**Published:** 2023-02-20

**Authors:** Roberta Carbonari Muzy, Aline de Piano Ganen, Marle dos Santos Alvarenga

**Affiliations:** I Centro Universitário São Camilo, Master's Program in Professional Nutrition – São Paulo (SP), Brazil.; II Centro Universitário São Camilo – São Paulo (SP), Brazil.; III Universidade Federal de São Paulo – São Paulo (SP), Brazil.; IV Universidade de São Paulo – São Paulo (SP), Brazil.

**Keywords:** Binge eating, Beliefs, Questionnaires, Adolescent, Compulsão alimentar, Crenças, Questionários, Adolescente

## Abstract

**Objective:**

The aim of this study was the cross-cultural adaptation of the Eating Belief Questionnaire (EBQ)—an instrument that assesses positive, negative, and permissive eating beliefs in relation to binge eating episodes—in its shorted version of 18 questions, into Brazilian Portuguese, for female teenagers.

**Methods:**

Conceptual, semantic, cultural and operational equivalence of the items were evaluated. The semantic and cultural equivalence involved 12 bilingual people and 12 experts in eating behavior. Operational equivalence consisted of applying the transcultural adapted version of the EBQ-18 to 20 girls with a mean age of 17.55 (SD=1.00) years. Item's clarity and understanding were assessed by the Content Validity Coefficient.

**Results:**

Questions 5, 6, 11, 14, 15, 16, 17 and 18, with adequate classification percentage for all equivalences, were not altered. The other items were adapted according to the committee's suggestions and by consensus among researchers. The adapted version of the EBQ-18 in Brazilian Portuguese displayed good content validity coefficient for clarity (CVC=0.975) and comprehension (CVC=0.971); except for item 3, all items had values between 0.88 and 1.00.

**Conclusion:**

The Brazilian Portuguese version of the EBQ-18 had a good understanding by the adolescent public when it comes to investigate the role of dietary beliefs in the maintenance of binge eating episodes. Future studies with adolescents are recommended, jointly assessing risk for and presence of eating disorders in significant clinical and non-clinical samples, as well as its psychometrics properties.

## RESUMO

**Objetivo:**

Conduzir adaptação transcultural, para o português brasileiro, da versão reduzida de 18 questões do *Eating Belief Questionnaire* (EBQ) ao público de meninas adolescentes. O instrumento avalia crenças positivas, negativas e permissivas do comer com relação a episódios de compulsão.

**Métodos:**

Realizou-se avaliação das equivalências conceitual, semântica, cultural e operacional dos itens. A equivalência semântica e cultural envolveu 12 bilíngues e 12 especialistas em comportamento alimentar. A equivalência operacional consistiu na aplicação da versão transcultural adaptada do EBQ-18 em 20 meninas, com média de idade de 17,55 anos (DP=1,00). O grau de clareza e compreensão das questões foi avaliado pelo coeficiente de validade de conteúdo.

**Resultados:**

Na avaliação semântica e cultural, 8 itens tiveram classificação adequada para todas as equivalências e não sofreram alterações; 10 itens foram alterados de forma mínima, conforme sugestões dos especialistas e mediante consenso entre pesquisadoras; e apenas o item 3 sofreu adaptação após resultados da equivalência operacional. O modelo adaptado para o português apresentou bom coeficiente de validade de conteúdo para clareza (CVC=0,975) e compreensão (CVC=0,971); com exceção do item 3, todos os itens foram avaliados com valores entre 0,88 e 1,00.

**Conclusão:**

A versão em português do EBQ-18 apresentou-se com boa compreensão do público adolescente para investigação do papel de crenças alimentares na manutenção de episódios de compulsão. Recomendam-se trabalhos futuros avaliando conjuntamente risco e presença de transtornos alimentares em amostras significativas clínicas e não clínicas, bem como suas propriedades psicométricas.

## INTRODUCTION

Eating behavior involves actions, cognitions and feelings related to eating, including beliefs, thoughts, and principles^1^. A common behavior related to binge eating episodes, a key characteristic of binge eating disorder (BED)^2^, is uncontrolled eating, described by the feeling of loss of self-control and/or inhibition of restraint behavior with consequent exaggerated consumption, with or without hunger or organic need^3,4^. Studies point out that eating disorders can persist based on the individual's beliefs about eating and food^5–10^.

Several psychological models for binge eating have been proposed in an attempt to better understand the BED, focusing on the role of dietary restriction, low self-esteem, low tolerance for suffering, excessive valuation of weight and body shape, and specific eating beliefs about binge eating^11^. Beliefs about food, which have no rational confirmation^12^, are related to several social, environmental and cultural factors, as well as socioeconomic level, education, family traditions, access to information about food that directly influence food choices^13^.

One of the cognitive processes related to restrictive diets that impacts the decision to give in to eating is compensatory beliefs: the view that the consequences of engaging in a behavior considered indulgent (eating a cake, a sweet, a dessert) by one who restricts “unhealthy” food or caloric intakes can be neutralized by another behavior (e.g., skipping dinner)^14^. The predictive validity of dietary beliefs was also observed in a longitudinal study, in which the expectation that eating would help control negative affection predicted the development of binge eating in female adolescent^5^.

Cooper et al.,^15^ emphasize the role of certain cognitive processes in maintaining binge eating episodes in a model that categorizes a set of beliefs considered positive about the role of compulsion in reducing emotional stress (“eating helps me cope”); permissive beliefs (“it's okay to binge eat”); and negative beliefs of “no control” (“once I start eating, I can't stop”)^15^. The most cited cognitive models to explain binge eating focus on the role of cognitive restriction, or the act of dieting^6,14,16^, in addition to low self-esteem and concern with body image, shape and weight^17–19^. However, few studies evaluate the cognitive processes that contribute to the maintenance of binge eating^15^.

The Eating Beliefs Questionnaire (EBQ-18) is an instrument used to assess positive, negative and permissive beliefs that maintain compulsive eating^20^ and, although other instruments are used to assess beliefs that act as triggers and/or to maintain eating disorders (ED), they focus on beliefs related to anorexia and bulimia nervosa, and not specifically on eating compulsion^21–24^. Most instruments available for evaluating binge eating focus on behaviors and diagnostic criteria rather than beliefs, or evaluate beliefs related to self-acceptance and control over eating^21–25^. Only the EBQ-18 had data validated in a clinical sample and investigates three dietary beliefs (positive, negative and permissive)^20^.

The EBQ was developed with input from experts in ED and cognitive behavioral therapy (CBT). It is a self-report measure, with originally 27 items addressing positive and negative beliefs that maintain binge eating^26^. Its factorial structure was explored in a clinical sample of university students in Australia, the community in general, people seeking treatment for ED and participants in a study on obesity^26^. Subsequently, through literature consultation and input from a committee of 10 experts, 19 permissive belief items were developed and included in the EBQ for online application to psychology students (n=767) and community participants (n=116). An exploratory factor analysis supported a three-factor solution (positive, negative, and permissive beliefs), explaining 63.4% of the variance, and a confirmatory factor analysis supported a shorted 18-item model (Cronbach's α=0.88 — negative beliefs; 0.92 — positive beliefs; and 0.88 — permissive beliefs)^20^. EBQ-18 scores correlated with frequency of binge eating episodes and increase in body mass index (BMI)^27^.

The EBQ-18's response options consider a 5-point Likert scale (from 1=totally disagree to 5=totally agree). Negative beliefs are related to negative convictions about eating (“I can't control my urge to eat”); positive beliefs, to the perceived benefits of binge eating (“eating makes me feel better”); and permissive beliefs refer to cognitive processes related to permissiveness (“I deserve it”, “it's okay to binge eat from time to time”)^20^.

Studies on the development and application of the EBQ^8,20,26–29^ supported its choice as a conceptually appropriate instrument for assessing dietary beliefs, especially for adolescents, who tend to show less engagement with more extensive instruments of evaluation.

Given the importance of metacognition in eating behavior^29^, the observation of a higher prevalence of compulsion among typical ED behaviors (restriction, compulsion and compensation)^30^, including national studies^31–37^, and the fact that studies that specifically assess eating beliefs are scarce, this study aims to cross-culturally adapt the EBQ-18 into Brazilian Portuguese to be used with female adolescents.

## METHODS

The cross-cultural adaptation process of the EBQ was based on universal recommendations^38,39^ regarding the items’ semantic, idiomatic, conceptual, cultural and operational equivalence. The authors of the original instrument were previously contacted and formally authorized the process of adaptation of the questionnaire into Brazilian Portuguese.

### Cross-cultural adaptation process

The conceptual equivalence stage consisted of exploring the construct of interest and assessing the relevance of the original instrument's dimensions, being carried out through a broad literature review on available instruments on dietary beliefs and adaptable to different target audiences.

The content equivalence step, aiming to guarantee that the adapted version would keep the same meaning of the original instrument, was carried out in translation steps; synthesis of translations; evaluation by expert and bilingual committees; and back-translation of final synthesized version.

The EBQ-18 was translated from the original English version into Brazilian Portuguese by two independent bilingual translators fluent and linguistically competent in both languages, but laypersons in the subject matter. The two translated versions were compared to define a single version (synthesis version 1).

The synthesis version 1 was submitted, with the original version in English, to a committee of 12 specialists in eating behavior (eight women and four men, with mean age of 31 years), comprising nine nutritionists, one physical education professional, a professor of psychology and a researcher who is also a dental surgeon, to evaluate the criteria of semantic equivalence (meaning of words in relation to vocabulary and grammar), idiomatic equivalence (adequacy of the meaning of expressions), cultural equivalence (adaptation to the cultural context of the target audience) and conceptual equivalence (maintenance of the originally proposed theoretical concept). Each question was evaluated and scored by the specialists, assigning “-1” for “Inadequate”; “0” for “Adequate” or “1” for “Extremely Adequate”^40^.

The synthesis version 2 was submitted to a committee of 12 bilingual people (eight women and four men, with mean age of 33.9 years), randomly divided into two groups to respond to the two versions of the questionnaire (in English and in Brazilian Portuguese) with an interval of five days, to verify agreement or incongruity in the understanding of the scale between languages. The paired t-test was performed to compare the responses of the bilingual sample to the original in English and the adapted version in Portuguese. The agreement between responses was evaluated by intraclass correlation coefficient (ICC)^41^, obtained through ratio between the variances of subjects and the sum of variances of subjects related to error. The ICC can range from zero to +1, with +1 being considered perfect^41^.

The adapted version was back-translated by a third translator and submitted to the authors of the original questionnaire for approval and confirmation of maintenance of the original meaning. After approval of the adapted version, the operational equivalence stage was carried out to assess the completion time, understanding and clarity of the questions by the target audience.

The questionnaire was completed in a digital form, available via Google Forms®, by a sample of 20 female adolescents enrolled in private schools in São Paulo. Adolescents aged between 14 and 18 years old, who presented the signed consent form by their guardians and assented their participation in the research, via Adobe Sign®, were eligible.

To calculate the content validity coefficient (CVC)^42^, to assess the clarity and understanding of the instrument, each item of the questionnaire was followed by a scale of 1 to 5 points, both for clarity and understanding (where 1=no clarity/understanding; and 5=complete clarity/understanding) and the minimum value accepted was 0.80^43^. The content validity coefficient was calculated as follows (Equation 1):


(1) $$\text{CVC}=\frac{\left(\text{sum of scores}÷\text{number of judges}\right)}{\text{Maximum value the item can be assigned}}$$

To remove possible biases of participants, the error calculation was performed (Equation 2):


(2) $$\text{Error}={\left(1÷\text{number of judges}\right)}^{\text{number of judges}}$$

So: CVC_final_=CVC − Error.

The students answered with their age, family income (alternative salary ranges according to minimum wages, based on Decree 9661/2019, which provides for the value of the minimum wage), parents’ education, self-reported weight and height, and school in which they were enrolled.

After applying the instrument to the selected sample and calculating the CVC, mean and standard deviation were obtained, followed by asymmetry and kurtosis measurements. Data were input in a Microsoft Excel® spreadsheet and later transferred for analysis in the R software (version 4.2 for Mac iOS).

This study was approved by the Research Ethics Committee, following the principles of the National Health Council (Resolution 466/2012) of the Ministry of Health for research with human beings.

## RESULTS

The main versions of the EBQ-18 during the cross-cultural adaptation process are presented in the supplementary material.

### Semantic, idiomatic, conceptual and cultural equivalence

After evaluating the specialists’ comments and classifying the equivalence of questions, changes were made based on the committee's suggestions and consensus between the researchers (Chart 1). Items 5, 6, 11, 14, 15, 16, 17 and 18 had an adequate percentage classification for equivalences, so no changes were needed. Questions 2, 3, 4, 7, 8, 9, 10, 12, and 13 underwent changes according to the committee's suggestions and consensus between the researchers.

**Chart 1:** Evaluation of equivalences by experts (n=12) of synthesis version 1 of the Eating Beliefs Questionnaire (EBQ-18).

Item	Semantic (%)			Idiomatic (%)			Conceptual (%)			Cultural (%)		
	I	A	EA	I	A	EA	I	A	EA	I	A	EA
1	8	25	67	8	58	33	8	33	58	17	17	67
2	0	17	83	0	25	75	0	33	67	0	33	67
3	8	25	67	17	33	50	67	17	17	33	33	33
4	8	8	83	0	8	92	0	8	92	0	8	92
5	0	8	92	0	0	100	0	0	100	0	8	92
6	0	0	100	0	8	92	0	17	83	0	8	92
7	0	17	83	0	27	83	17	27	67	8	25	67
8	0	8	92	0	8	92	0	17	83	0	8	92
9	8	17	75	8	17	75	17	33	50	17	8	75
10	0	8	92	0	17	83	0	17	83	0	17	83
11	0	0	100	0	0	100	0	0	100	0	8	92
12	8	33	58	0	33	67	17	42	42	8	50	42
13	0	17	83	0	17	83	0	17	83	8	8	83
14	0	0	100	0	0	100	0	0	100	0	0	100
15	0	0	100	0	0	100	0	0	100	0	0	100
16	0	0	100	0	0	100	0	0	100	0	0	100
17	0	0	100	0	0	100	0	8	92	0	8	92
18	0	0	100	0	0	100	8	17	75	8	8	83

I: Inadequate; A: Adequate; EA: Extremely Adequate.

Although question 1 was assessed as 8% “Inadequate” regarding semantic, idiomatic and conceptual equivalence, 17% regarding conceptual equivalence because of the English term “urge to eat”, suggestions and comments were divergent regarding the best term to be used in the translation.

In question 2, “means” was changed to “is a form of”. In question 3, “só (only)” was included to denote the secrecy of compulsion. In question 4, the adverb “when” was changed to “since”. In question 7, the pronoun “I” was added at the beginning of the sentence. In question 8, “more” was added before the word “bearable”. The expression “all right” in question 9 was replaced by “no problem”. In question 10, “my diet” was changed to “my way of eating”. In question 12, “the compulsion” was changed to “compulsive eating”. The sentence in question 13 “I will never stop eating” was changed to “I never stop eating”.

The synthesis version 2, with these adaptations, was submitted to a committee of 12 bilingual people, randomly divided into two groups to respond to both versions (original and synthesis 2), with an interval of five days. The comparison of responses for the English and Brazilian Portuguese versions is shown in Table 1. There was no significant difference between mean responses for any of the items between versions. The question with the lowest agreement was item 7, “I have no willpower when it comes to food”, and the question with the highest agreement was item 3, “Compulsive eating is something I can have all to myself”. After analyzing the bilingual evaluators’ answers, some questions were discussed again with eight of them to check any difficulties in interpretation or any other reason for different answers to the same questions in different versions. Most of them reported different scores because they responded to the versions at different times.

**Table 1:** Comparison of scores of bilingual responses (n=12) to questions in the original and adapted versions of the Eating Beliefs Questionnaire (EBQ-18).

Questions	Mean in Portuguese (SD)	Mean in English (SD)	p-value	ICC [95%CI]	SEm
Q1	1.83 (0.718)	2.17 (1.11)	0.256	0.538 [0.114–0.804]	0.6276
Q2	2.58 (1.51)	2.25 (1.29)	0.548	0.486 [0.293–0.780]	1
Q3	2.25 (1.215)	2.58 (1.240)	0.072	0.893 [0.670–0.963]	0.3482
Q4	2.33 (1.155)	2.17 (1.403)	0.572	0.797 [0.531–0.922]	0.5774
Q5	3.33 (1.155)	2.92 (1.240)	0.120	0.749 [0.427–0.903]	0.5607
Q6	2.25 (1.215)	2.33 (1.303)	1.000	0.761 [0.462–0.907]	0.6124
Q7	1.67 (0.651)	1.50 (0.522)	0.586	0.275 [-0.220–0.662]	0.5
Q8	3.00 (1.279)	2.42 (1.240)	0.106	0.585 [0.182–0.826]	0.7662
Q9	1.75 (1.138)	2.08 (1.240)	0.330	0.651 [0.275–0.858]	0.6963
Q10	2.00 (0.953)	1.67 (0.778)	0.182	0.577 [0.173–0.822]	0.5505
Q11	2.83 (1.467)	2.92 (1.279)	0.890	0.772 [0.481–0.912]	0.677
Q12	1.75 (0.965)	2.42 (1.379)	0.054	0.583 [0.125–0.827]	0.6963
Q13	2.50 (1.382)	2.25 (1.422)	0.586	0.593 [0.180–0.832]	0.8898
Q14	3.17 (1.528)	2.58 (1.240)	0.089	0.739 [0.373–0.901]	0.6366
Q15	1.58 (0.900)	1.75 (1.055)	0.586	0.739 [0.421–0.898]	0.5
Q16	1.67 (0.888)	1.25 (0.452)	0.120	0.327 [0.098–0.678]	0.5607
Q17	1.92 (0.900)	2.25 (1.055)	0.410	0.307 [-0.185–0.681]	0.8165
Q18	1.75 (1.055)	2.42 (1.240)	0.098	0.500 [0.076–0.781]	0.7588

ICC: intraclass correlation coefficient; 95%CI: 95% confidence interval; SEm: standard error of measurement.

The observations made by the bilingual evaluators were discussed, and questions 9, 13 and 18 were again submitted to the expert committee for evaluation of possible final adaptations. For question 9, an option in Brazilian Portuguese was requested for the English expression “experience of binge eating” (“*experiência comendo compulsivamente*”; “*experiência de comer compulsivamente*”; “*experiência de compulsão*”). For question 13, “If I don't control myself, I never stop eating”, we inquired about the most appropriate tense between “If I don't control myself, I never stop eating” and “I can't stop eating”. For question 18, “I like to binge”, we requested the best option between “I like to eat compulsively” and “I like to eat compulsively”. Question 1, which brings the term “urge to eat”, was also discussed as for the term in Portuguese, to express will, desire or food impulses.

After this last review by specialists, which resulted in the synthesis version 2 of the EBQ-18, the questionnaire was submitted to back-translation and then for approval by the authors of the original questionnaire. They approved the version adapted to Brazilian Portuguese and confirmed that the meaning and intention of the original EBQ-18 were maintained in the adaptation process, reinforcing that it be pointed out, in the discussion of any publication that presents this translation, that the intention is that the items assess “binge eating” according to the criteria described in the DSM-5^1^.

The synthesis version 2 was applied to the target audience aiming to assess operational equivalence. The participants (n=20) were aged between 14 and 18 years old (mean=17.55, SD=1.00); their mean weight was 65.97 kg (SD=8.62), mean height was 1.65 m (SD=0.05), mean BMI was 24.11 kg/m^2^ (SD=2.91); and mean Z-score of 0.70 (SD=0.66). As for family income, 10% of the sample reported an income of up to 2 minimum wages; 15%, between 2 and 4 minimum wages; 20%, between 4 and 10 minimum wages; and 55% reported more than 10 minimum wages (data not reported in the table).

The adolescents took, on average, 4.4 minutes (SD=1.23 minutes) to answer the EBQ-18. The average score of each item, respective standard deviations and measures of asymmetry and kurtosis are listed in Table 2.

**Table 2:** Descriptive characterization of the Eating Beliefs Questionnaire (EBQ-18) obtained by the adolescents (n=20).

Item	Mean	Median	Standard deviation	Asymmetry	Kurtosis
1	2.60	2.0	1.10	0.10	–1.48
2	2.40	2.0	1.39	0.75	–0.77
3	2.50	3.0	1.19	0.09	–1.01
4	2.25	2.0	1.21	0.39	–1.48
5	2.60	2.0	1.14	0.37	–0.96
6	1.80	1.5	1.01	0.97	–0.30
7	2.25	2.0	1.07	0.75	0.07
8	2.75	3.0	1.29	–0.11	–1.42
9	1.65	1.0	0.88	1.13	0.34
10	2.35	2.0	1.04	0.38	–1.14
11	2.30	2.0	1.38	0.51	–1.37
12	1.75	2.0	0.91	1.27	0.94
13	2.70	2.0	1.56	0.32	–1.55
14	2.70	2.0	1.30	0.13	–1.55
15	1.70	1.5	0.86	1.03	0.24
16	1.60	1.0	0.82	1.32	1.26
17	2.00	2.0	0.92	0.39	–1.03
18	1.30	1.0	0.57	1.58	1.43

The clarity grade for the EBQ-18 had a CVCt of 0.975. With the exception of item 3 (CVC=0.79), all items were above the established cutoff point^43^. As for understanding in the EBQ-18, the CVCt was 0.97, and all items were evaluated with values between 0.88 and 1.00 (Table 3), except item 3 (CVC=0.72).

**Table 3:** Assessment of the level of clarity and understanding of the Eating Beliefs Questionnaire (EBQ-18) by adolescents (n=20).

Item	Clarity			Understanding		
	Mean	Standard Deviation	CVC	Mean	Standard Deviation	CVC
1	4.95	0.22	0.99	4.90	0.45	0.98
2	5.00	0.00	1.00	5.00	0.00	1.00
3	3.95	1.28	0.79	3.60	1.57	0.72
4	5.00	0.00	1.00	5.00	0.00	1.00
5	5.00	0.00	1.00	5.00	0.00	1.00
6	4.85	0.49	0.97	4.75	0.55	0.95
7	4.40	0.94	0.88	4.40	0.88	0.88
8	5.00	0.00	1.00	5.00	0.00	1.00
9	5.00	0.00	1.00	5.00	0.00	1.00
10	4.95	0.22	0.99	5.00	0.00	1.00
11	5.00	0.00	1.00	5.00	0.00	1.00
12	4.90	0.31	0.98	4.90	0.31	0.98
13	5.00	0.00	1.00	5.00	0.00	1.00
14	5.00	0.00	1.00	5.00	0.00	1.00
15	4.95	0.22	0.99	4.95	0.22	0.99
16	5.00	0.00	1.00	5.00	0.00	1.00
17	5.00	0.00	1.00	5.00	0.00	1.00
18	4.85	0.49	0.97	4.90	0.45	0.98

CVC: content validity coefficient.

One participant reported difficulty understanding question 3 (“Compulsive eating is something I can have all to myself”); another one reported being in doubt about the clarity of question 7 (“I don't have any willpower when it comes to food”); and another one described difficulty with questions 3 and 18 (“I like to binge eat.”). The others reported not having difficulty understanding any of the 18 questions.

After evaluating the results, discussing with specialists and confirming the conceptual adequacy of item 3 of the version adapted by the authors of the original instrument, item 3 of the permissive eating beliefs scale was adjusted to “Compulsive eating is something I can have/do just for me”. The item also had the highest ICC (0.893) among all the items when comparing the scores of the bilingual answers to the questions in the original and adapted versions, reinforcing the agreement between the versions and the decision to maintain the item in adequate conformity with the original version (final version of supplementary material).

## DISCUSSION

This study achieved the objective of conducting the cross-cultural adaptation of the reduced version of 18 questions of the EBQ into Brazilian Portuguese, aimed at female adolescent, following methodological guidelines. In addition to classic recommendations^38,39^, this process included the application of the questionnaire to a bilingual committee to analyze the agreement between versions, approval of the adapted version by the authors of the original instrument.

The adapted instrument assesses dietary beliefs that maintain episodes of binge eating, an important study for the field of nutrition, especially due to the high prevalence of risk for binge eating in Brazilian adolescents^31,44^. So far, there are no studies evaluating this aspect of eating compulsion—maintenance beliefs—in Brazil, and even internationally, studies are scarce. Regarding the studies that used the EBQ-18, due to the recent development, there are only studies with adults^8,20,26–29^. This work is pioneer in presenting a new tool for assessing eating-related beliefs in the Brazilian culture, focused on female adolescents, who are especially more susceptible to eating disorders^1,45^.

The cross-cultural adaptation of the EBQ-18 resulted in an instrument that is easy to use, with satisfactory results in terms of clarity and understanding, and the sample that participated in this evaluation showed characteristics similar to those of the Brazilian population in this age group^46^, in which the majority is eutrophic, with adequate height for age, although a high socioeconomic level was observed.

We emphasize the possibility of using this instrument with boys, since minimal adjustments would be necessary (gender pronouns), as observed in previous studies, with the application of the EBQ-18 in other countries for both men and women^8,20,26–29^. The adapted version does not present any application restriction for adults of the same culture, since it fully kept the concept of the original scale items.

One should consider limiting the use of a convenience sample. However, in the operational equivalence stage, there is no requirement for such a broad and heterogeneous public, as it is intended for the assessment of psychometric properties^38,43^. One difficulty in the process, pointed out by the specialists and by the bilingual committee, was the existence of terms in English without a literal translation into Brazilian Portuguese, such as the term “binge”, which, in English, is also used as a verb, not having a single word that could replace the term. In order to maintain the instrument's original concept, the terms were adapted to expressions describing binge eating according to the DSM-5^1^.

In order for the EBQ-18 to be used in evaluation studies and epidemiological and clinical protocols focused on eating beliefs and compulsion, it is necessary that new studies evaluate the psychometric properties in each target audience^41,42^. It is recommended that further studies explore correlations between the EBQ-18 factors and concerns related to body shape, since previous studies highlight their relevance in eating behaviors among adolescents, with significant paths to compulsive eating^19,45,47^. Studies carried out with the EBQ-18 reported a negative correlation of its subscales with self-compassion, self-esteem and food self-efficacy, central issues studied in binge eating^8,29^.

After the cross-cultural adaptation, we concluded that the instrument satisfactorily meets the conceptual, idiomatic, semantic, cultural and operational equivalences and is suitable and available for use in a population of Brazilian adolescents.
